# Effect of aerobically treated manure on odorous material emissions from a swine finishing barn equipped with a continuous pit recirculation system

**DOI:** 10.5713/ab.21.0135

**Published:** 2021-06-23

**Authors:** Yongjun Choi, Duck-Min Ha, Sangrak Lee, Doo-Hwan Kim

**Affiliations:** 1 Department of Animal Science and Technology, Konkuk University, Seoul 05029, Korea; 2 Department of Animal Resources Technology, Gyeongnam National University of Science and Technology, Jinju 52725, Korea

**Keywords:** Aerobic Digestion, Continuous Pit Recirculation System, Liquid Fertilizer, Odorous Material, Swine Facility

## Abstract

**Objective:**

This study was conducted to determine reduction of various odorous materials from a swine farm equipped with a continuous pit recirculation system (CPRS) with aerobically treated liquid manure.

**Methods:**

The CPRS is used in swine farms in South Korea, primarily to improve air quality in pig houses. In this study, CPRS consists of a manure aerobic treatment system and a fit recirculation system; the solid fraction is separated and composted, whereas the aerobically treated liquid fraction (290.0%±21.0% per day of total stored swine slurry) is continuously returned to the pit. Four confinement pig barns in three piggery farms were used; two were equipped with CPRS and the other two operated a slurry pit under the slatted floor.

**Results:**

All chemical contents of slurry pit manure in the control were greater than those of slurry pit manure in the CRPS treatment (p<0.05). Electrical conductivity and pH contents did not differ among treatments. The biological oxygen demand of the slurry pit treatment was greater than that of the other treatments (p<0.05). Total nitrogen, total phosphorus, and ammonia nitrogen contents of the slurry pit treatment were greater than those of other treatments (p<0.05). Odor intensity of the CPRS treatment was lower than that of the control at indoor, exhaust, and outside sampling points (p<0.05). The temperature and carbon dioxide of the CPRS treatment in the pig barn was significantly lower than those of control (p<0.05). All measured odorous material contents of the CPRS group were significantly lower than those of the control group (p<0.05).

**Conclusion:**

The CPRS application in pig farms is considered a good option as it continuously reduces the organic load of animal manure and lowers the average odorant concentration below the threshold of detecting odorous materials.

## INTRODUCTION

The livestock industry has grown tremendously, causing significant manure emissions and negative effects on the environment. Livestock excretes manure, which releases various compounds including hydrogen sulfide (H_2_S), ammonia (NH_3_), odorous volatile organic compounds (VOCs), and volatile inorganic compounds (VICs). Gaseous materials from swine manure cause not only various environmental pollutants but also respiratory discomfort and the depression of growth in pigs [1]. The main odorous compounds, which occurred from swine manure, were classified as volatile fatty acids (VFA), phenols, volatile sulfur-containing compounds, and volatile amines [2]. H_2_S is produced from manure under anaerobic conditions, and it has a strong odor at a very low concentration. Furthermore, high H_2_S levels in the air are strongly correlated with the deaths of livestock animals [3]. Recently, NH_3_ has been recognized as an important contributor to the formation of particulate matter such as PM_2.5_, which causes air pollution and health problems [4,5]. Phenolic compounds and indoles have a low odor threshold and are widely spread in swine facilities [6]. The occurrence of these odorous substances has raised concern about the increasingly negative impacts on the livestock industry. Due to this there is an increasing number of swine farms using continuous pit recirculation systems (CPRS) to improve indoor air quality and reduce the odor of swine facilities such as NH_3_ and hydrogen sulfide, in South Korea [7]. Aerobically treated liquid swine manure is continuously recharged into the slurry pit using a pump system. This system can be expected to reduce gas emissions due to the dilution of raw swine manure by aerobically treated liquid manure [8]. It has been reported that H_2_S and NH_3_ emissions in finishing pig housing equipped with a semi-continuous pit recharge system can be reduced by 53% and 84%, respectively [7]. Although the effects of H_2_S and NH_3_ reduction have been reported in swine farms equipped with a CPRS, research is rare on odorous materials such as nitrogen compounds, sulfur compounds, and VOCs. Therefore, this study was conducted to determine the reduction of NH_3_, H_2_S, nitrogen compounds, sulfur compounds, and VOCs from a swine farm equipped with CPRS and aerobically treated liquid manure.

## MATERIALS AND METHODS

### Farm description, continuous pit recirculation system, and experimental design

**Figure 1:**
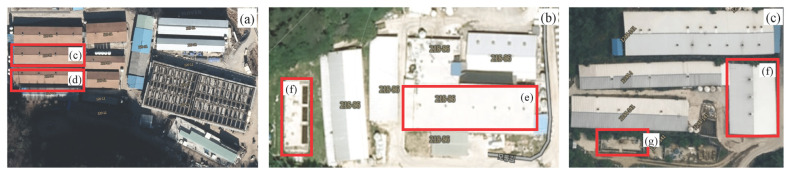
Pictures of experimental farms. (a) a common farm not equipped with continuous pit recirculation system (Farm A, control); (b) Farm B; and (c) Farm C. (d) and (e) two rooms of a finishing pig confinement building in a farm not equipped with continuous pit recirculation system; (f) and (g) two room of a finishing pig confinement building in the farm equipped with continuous pit recirculation system; (h) and (i) air-treated liquid manure manufacturing facility.

**Figure 2:**
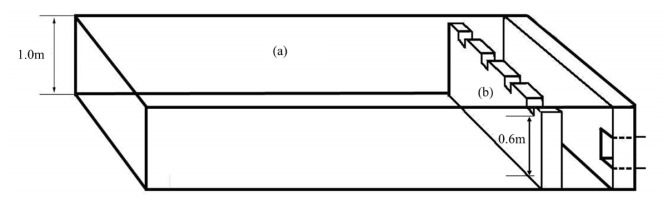
Concrete pit of the pig barn. (a) Slurry pit; (b) Stank.

**Figure 3:**
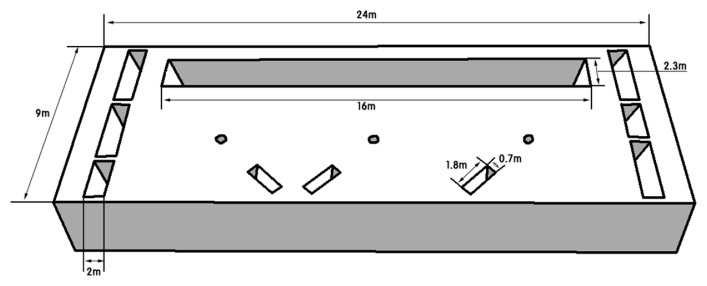
Dimensions of the thermal mesophilic aerobic digestion facility.

**Figure 4:**
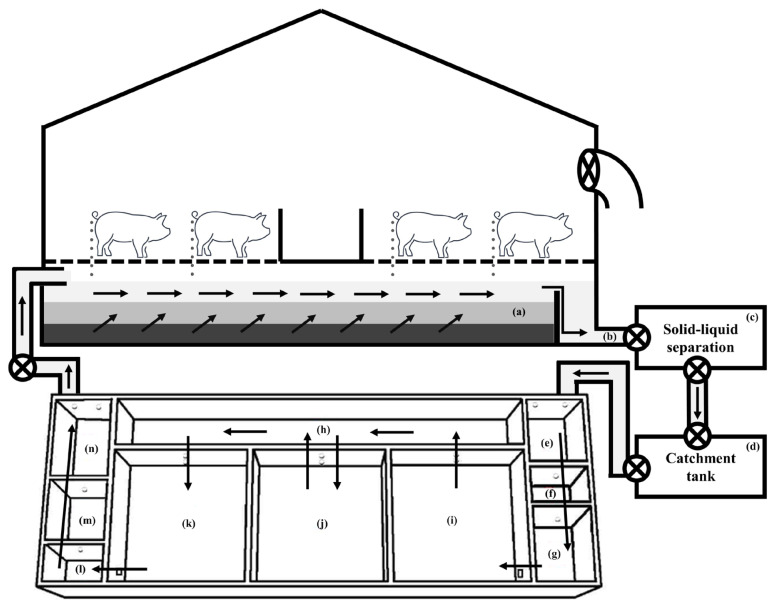
Schematic diagram of the experimental farm facilities with a continuous pit recirculation system and the auto thermal mesophilic aerobic digestion facility. (a) pit mixing; (b) slurry outlet; (e) slurry tank; (f) flow control tank; (g) inlet tank; (h) first aeration tank; (i) anoxic (first and second); (j) second aeration tank; (k) third aeration tank; (l) first settling tank; (m) second settling tank; (n) circulation tank.

**Figure 5:**
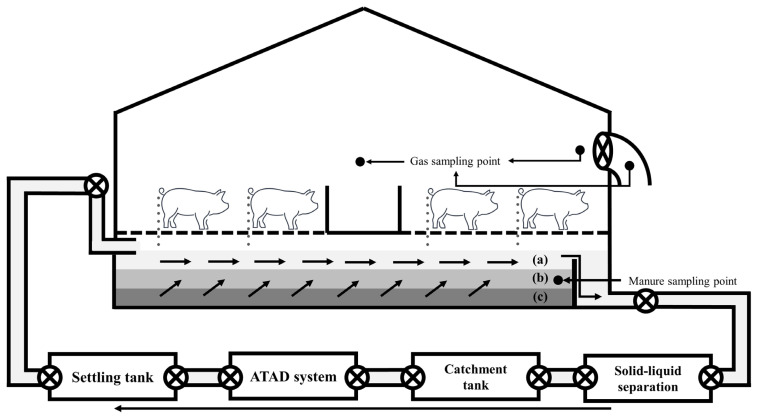
Schematic diagram of the experimental farm facilities with a continuous pit recirculation system (a) 40 to 60 cm; (b) 20 to 40 cm; (c) under 0 to 20 cm; (d) indoor gas sampling point; (e) exhaust fan sampling point.

The experiment was carried out at each finishing pig confinement building of three commercial swine farms in South Korea (Table 1; Figure 1) for one year. The confinement buildings for finishing pigs, which consisted of one room at each farm, were used to evaluate the effect of a CPRS on inner odorous materials. Two room ([d] and [e]; Figure 1) with only a slurry pit was used as a control (Farm A [a]), and the other two rooms ([f] and [g]; Figure 1; Farm B [b] and Farm C [c]) used had a slurry pit equipped with a stank (Figure 2), in which the pit continuously recirculated 148 kg per head of pig aerobically treated liquid manure every day, approximately 29 times the daily production of manure from pigs. The amount of recirculation amount was designed based on the assumption that 5.1 kg of combined manure, urine, and washing water excreted daily per head of pig on Farms B and C according to a basic unit of livestock manure discharge in South Korea. The total amount of recirculated aerobically treated liquid manure in a day was roughly 290.0% ±21.0% (29.0±2.1 times per day) of total stored swine slurry in the pit. Swine manure and recirculated liquid manure were maintained at a constant depth (60 cm) throughout the experimental period. The recirculation process was divided into twelve steps: pit mixing, slurry out, separation, catchment, flow control, first aeration, first anoxic, second aeration, second anoxic, third aeration, settling, storage, and recirculation (Figures 3, 4). The mixture of manure and recirculated liquid continuously existed in a fixed quantity (approximately 60% of the total pit volume) in the pit, and the manure was continuously excreted by liquid recirculation from the house. The treated liquid manure is considered a “liquid fertilizer” in CPRS and is periodically stored into a storage tank and then spread to cropland during the period specified by law in South Korea. Each experimental barn allocated finishing pigs, and the average stocking density was 0.92±0.18 m^2^/head (Farm A, 1.00 m^2^/head; Farm B, 1.05 m^2^/head; Farm C, 0.72 m^2^/head). The barns consisted of a fully slatted floor, and manure pits were 1.0 m deep and had 0.6 m shank (Figure 2). The recirculation liquid manure was sampled in April, August, October, and November for 2 years at 1000 to 1200 h. Samples were collected from the storage tank, slurry pit under swine room, outlet of slurry pit, and anoxic tank (Figure 4). Samples from the slurry pit under the swine room were collected at heights of 30 cm (Figure 5; middle) from the bottom of the pit where recirculation liquid and pig manure were mixed (Figure 3). The temperature, humidity, and wind velocity inside the barn were measured at the same time as the manure sample collection. The air ventilation system was separated in each room and managed independently. The air inlets were in the ceiling (Farm A, four air inlets; Farm B, six air inlets; Farm C, three air inlets) and exhaust ventilation fans were wall-mounted. The air sampling locations of each room were central points in the room, downstream and upstream of each continuously operated fan, and outside of the room (Figure 5).

### Odorous materials

Air samples were collected using a gas sampling box (Cos-100, Kemic Co., Sungnam, Korea) equipped with a 20 L air sampling aluminum bag (TD-AP20, Whirl-Pak, WI, USA) at a height of 5 m, and sampling was carried out using an inner finishing pig barn, exhaust fan, and the site boundary of the farm. Air samples transported using a light-resistant container at a range of 15°C to 25°C. The air sensory test was performed within 48 h after sample collection. Temperature, humidity, and wind velocity of indoor atmospheres were measured using a digital anemometer assembly (Testo 410-2, Testo SE & CO., Lenzkirch, Germany) inside the experimental room (Figure 5). All odorous materials were analyzed according to the standard method for odor estimation in South Korea [9]. Odor intensity was scored according to a 6-ladder whole number scale using the air dilution sensory method of the standard methods for the examination of odor [9] (0, undetectable; 1, barely detectable; 2, moderate; 3, strong; 4, very strong; 5, unbearable); it was determined using average value of 5 expert members. NH_3_ was determined according to a modified colorimetric method for the determination of NH_3_ in the air with phenol and sodium hypochlorite [10] using a spectrophotometer (Cary 300 UV-Vis; Agilent Technologies, Santa Clara, CA, USA) at 640 nm. The sulfur compounds (H2S, methyl mercaptan [MM], and dimethyl disulfide [DMD]) were concentrated using a sulfur compound analyzer (Unity/Air Server XR, Markers International, Bridgend, Wales, UK), and sulfur compounds were analyzed using gas chromatography (HP 6890; Agilent Technologies, CA, USA) [11]. Helium gas and flame photometric detectors were used as the carrier gas and detector, respectively. Air was collected according to the impinger method [12] for the analysis of trimethylamine (TMA). TMA was determined using gas chromatography equipped with a thermal desorption–cryofocusing system according to the standard method for odor estimation in South Korea [9]. Phenol, indole, and skatole were determined using gas chromatography equipped with a solid-phase microextraction filter [13].

### Manure analysis

Manure samples were stored below −20°C and analyzed for pH, electrical conductivity (EC), biological oxygen demand (BOD), chemical oxygen demand (COD), suspended solids (SS), total nitrogen (T-N), total phosphorus (T-P), ammonium nitrogen (NH_4_-N), total organic carbon (TOC), and total carbon (TC). Biological oxygen demand, COD, SS, T-N, and T-P were analyzed using the standard American Public Health Association (APHA) method [14]. The pH was determined using a digital pH meter (Orion 4 Star, Thermo Scientific, Waltham, MA, USA), and EC was determined using a conductivity meter equipped with a real-time data logger (YK 2005CD, Lutron Electronic Enterprise Co., Taipei, Taiwan). The T-N content in manure was measured using the Kjeldahl method [15]. NH_4_-N was determined according to the method of Chaney and Marbach [16]. The TC and TOC were analyzed using a total organic carbon analyzer (TOC-L, Shimadzu Corporation, Kyoto, Japan).

### Statistical analysis

Data were analyzed using the MIXED procedure of the SAS package program (SAS Inst. Inc., Cary, NC, USA) as a completely randomized software. The model was,


$$\text{Yi}(\text{t})=\mu +{\text{T}}_{\text{i}}+{\text{B}}_{\text{j}}+{\text{E}}_{\text{ij}(\text{t})},$$

where μ is the average value, T_i_ is the treatment value, B_j_ is a random value, and E_ij(t)_ is the error value. The model included CPRS as the fixed effect and season as the random effect. Pairwise comparisons were performed to determine the CPRS effect using the TTEST option. Orthogonal contrasts were used to determine the CPRS effect, location effect, and interaction between the CPRS and location effect using the CONTRAST option. Least squares mean values were assessed using a pairwise comparison method and the orthogonal contrast method. Treatment effects were considered significant at p<0.05, and trends were considered at 0.05≤p<0.10.

## RESULTS

The chemical properties of the slurry pit manure according to the application of a CPRS in the finishing pig house are shown in Table 2. The EC, pH, BOD, COD, SS, T-N, T-P, NH_4_-N, TOC, and TC contents showed highly significant differences between the treatments (p<0.05). The chemical content of slurry pit manure in the control was greater than that of slurry pit manure in the CRPS treatment (p<0.05).

The chemical properties of the recirculation liquid by the treatment process equipped with the CPRS are listed in Table 3. The EC and pH contents did not differ among treatments. The BOD showed significant differences among the treatments (p<0.05), and the BOD content of the slurry pit treatment was greater than that of the other treatments (p<0.05). The COD and SS contents did not differ among treatments. The T-N, T-P, and NH_4_-N contents were significantly different among the treatments (p<0.05), and the T-N, T-P, and NH_4_-N contents of the slurry pit treatment were greater than those of the other treatments (p<0.05). TOC and TC contents did not differ among the treatments.

The odor intensity and atmospheric characteristics in the finishing pig house according to the application of the CPRS are shown in Table 4. The odor intensity of the CPRS treatment was lower than that of the control at indoor, exhaust, and outside sampling points (p<0.05). The indoor atmosphere, temperature, CO_2_, NH_3_, and H_2_S contents of the CPRS treatment were significantly lower than those of the control (p<0.05). Humidity and wind velocity did not differ between the control and CPRS treatments.

The comparison of odorous material contents at various sampling points according to the application of the CPRS is shown in Table 5. NH_3_, H_2_S, MM, DMD, TMA, phenol, indole, and skatole content showed significant differences and interactions among the treatments (p<0.05). All measured odorous material contents showed that the CPRS group was significantly lower than the control group (p<0.05), and the odorous material contents of indoor and exhaust were significantly greater than those of the outside (p<0.05).

## DISCUSSION

In this study, all chemical properties of the CPRS group were lower than those of the control group. In a previous study, pig slurry was reported to have a COD content of approximately 8,400 to 100,000 mg/kg and a nitrogen content of 520 to 8,800 mg/kg [17,18]. In this study, the COD of manure in the pit showed that manure excreted in the pig house was continuously diluted by the air-treated liquid manure. Feed rate is a critical control component when operating aerobic digestion owing to the possibility of organic loading shock [19]. Although a high organic loading rate does not necessarily degrade the organic matter degradability, it causes the digester to become unstable. Further, aerobic granules are made well at a low organic loading rate [20]. In CPRS, as the diluted slurry is continuously discharged, the organic loading rate decreases the inflow to the aerobic digester and thus has a positive effect on the stability of aerobic digestion. Furthermore, the application of a pit recirculation system causes a decrease in the T-N and NH_4_-N content in the slurry, thereby reducing NH_3_ in the air of the pig house [21]. Although the BOD content of the anoxic tank was the lowest during the recirculation process, those of the storage tank and outlet of the slurry pit were maintained at a lower level than that of the slurry pit through dilution and degradation during the recirculation process. In this study, nitrogen was removed through nitrification and denitrification during the anoxic process [22]. It could be explained that the T-N and NH_4_-N contents of the slurry pit were greater than those of the anoxic and storage tanks. In this study, since the chemical properties of the pit manure decreased by the recirculation of air-treated liquid manure, it might be considered that the odor intensity and indoor atmosphere improved (Table 4). Odor intensity in the CPRS group was 36.4%, 41.4%, and 51.4% lower than that of the control group in the indoor, exhaust, and outside samples, respectively. Many odorous materials of pig slurry occur in the air during aerobic digestion, which means that the amount of odorous materials decreases in aerobic-treated liquid manure. Furthermore, as aerobic treated liquid manure continuously flows to the upper layer of slurry in the pit, it has two effects: it dilutes the slurry with high organic matter content and blocks the odor generated from the surface of the slurry [21]. In this study, as the fact that the wind velocity of the exhaust fan did not differ between the control and CPRS groups, it can be considered that CPRS has the ability to control temperature compared to the control group. Furthermore, in a previous study, it was reported that the room temperature of the Autothermal thermophilic aerobic digestion (ATAD) pit recirculation system was significantly lower than that of the control group at the same ventilation rate and under the same animal stock density conditions [7]. In this study, the difference that can affect the environmental conditions was the animal stock density in the pig house between the treatment and control groups. However, it is considered that the temperature change due to animal stock density will be very small. Although the effect of CPRS on room temperature was compounded with the animal stock density of pig houses, a previous study [7] and this study suggested the possibility that CPRS could control room temperature. The effect of CPRS on NH_3_ reduction in pig house was reported by similarly themed papers by the same author and showed opposite results [7,21]. A previous study explained that the high protein feed fed to finishing pigs in the previous study led to a nitrogen flux in the recirculating liquid, finally generating NH_3_ at high levels in the ATAD pit recirculation system, specifically during the summer season [7]. This suggests that NH_3_ in the pig house can be effectively reduced by controlling the amount of nitrogen supplied to the aeration tank. In this study, the equipped CPRS was a continuous type, i.e., it can continuously lower the content of organic matter flowing into the aeration tank by dilution of slurry and help to maintain low nitrogen flux during the aerobic process. Conversely, if air-treated liquid manure, which nitrogen is not sufficiently removed using the denitrification process, is inputted into the slurry pit, there is a risk of an increase in ammonia in the pig house. Therefore, the application of CPRS to piggery farms and continuous care about nitrogen is expected to reduce NH_3_ emissions from the slurry in the pit. Generally, H_2_S is generated under anaerobic conditions, with generation being increased under low pH conditions compared to that under high pH condition and with the main source being derived from proteins [23]. In this study, the pH of CPRS was lower than that of the control. However, the difference in pH values was small compared with that in a previous study [7,21]. In contrast, the reduction effect of H_2_S in the CPRS was similar to that observed in previous studies. It considers that there are two possibilities. One is considered that is the action of inhibiting anaerobic microorganisms that produce hydrogen sulfide by recirculating aerobic-treated liquid manure into a slurry pit [24] and another one is that is considered to be a blocking effect of odor materials emission from slurry due to the air-treated liquid manure flowing at the top of the pit; most importantly, this study showed that H_2_S can be reduced regardless of the pH value through the application of CPRS in pig farms. Most odorous materials are generated by microorganisms in the slurry pit. The several aims of CPRS are i) to shield odor materials coming up from the bottom by continuously flowing liquid to the top of the pit, ii) Lowering the concentration of organic matter in the pit by continuously discharging a portion of the pit manure, and iii) to increase the number of useful microorganisms instead of odor-producing microorganisms by supplying air-treated liquid manure. In this study, various odorous materials from the pig slurry pit were reduced in the CPRS group compared with those in the control and it showed the evidence that the application of CPRS in a pig house showed the reduction effect on a wide range of odorous materials. However, although CPRS is very useful for reducing odorous material from pig houses and slurry, there are still many problems to be solved. The long-term operation of CPRS can lead to problems such as the clogging of valves by salts. These problems could be solved through solid-liquid separation, advancement of the sedimentation process, and installation of strainers. In addition, the problems are caused by lack of management such as microbial imbalance, input high concentrations of nitrogen in the pit, and accumulation of the solidified slurry in the pit. These problems can worsen the odor problem in the pig house, so must be more careful. Apart from the reduction of odorous material in the pit using CPRS, there is a clear possibility that many odorous materials could still be generated in the process of manufacturing liquid manure and composting separated solids from the slurry. Nevertheless, the application of CPRS in swine farms is considered a good option as it continuously reduces the organic load of animal manure and lowers the average odorant concentration below the threshold.

## CONCLUSION

A CPRS with air-treated liquid manure effectively reduced ammonia, hydrogen sulfide, nitrogen compounds, and VOCs emission from pig houses. The chemical composition of properties, such as BOD and COD, was significantly lower in the equipped CPRS farm than in the unequipped farm. The application of CPRS in swine farms could considered to be viable because the system continuously reduces the organic load of animal manure and lowers the average odorant concentration below the threshold of detecting odorous materials.

## Figures and Tables

**Table 1:** Condition of experimental swine house

Farm^1)^	Total number of pigs	Housing type	Ventilation	Number of pigs in the barn	Housing size (m)	Fermenter size (m)	Storage tank (m)	Catchment tank (m)
A	4,000	Confined	Forced	600	50×12×2.7	18×8×2	42×13×2	-
		Confined	Forced	600	50×12×2.7	18×8×2	42×13×2	-
B	3,000	Confined	Forced	600	35×18×4	21×8×3	7×7×4.5	30×7×3
C	2,000	Confined	Forced	400	22×13×3	24×9×3	25×2×3	12×7×3

1) A, a common farm not equipped with continuous pit recirculation system; B and C, farms operating continuous pit recirculation systems (Figure 1).

**Table 2:** Comparison of chemical properties of slurry pit manure according to the application of a continuous pit recirculation system in the finishing pig house

Items	Control	CPRS	SEM	p-value
EC (dS/m)	24.1	12.6	3.32	<0.001
pH	7.7	7.9	0.07	0.011
BOD (mg/L)	14,157.0	1,563.4	1,237.4	<0.001
COD (mg/L)	18,541.0	3,319.8	2,143.7	<0.001
SS (mg/L)	35,063.0	3,425.6	5,261.2	0.001
T-N (mg/L)	5,786.5	1,153.7	575.5	<0.001
T-P (mg/L)	1,444.3	150.4	346.3	0.006
NH_4_-N (mg/L)	2,003.3	606.5	72.8	<0.001
TOC (g/L)	12,214.0	2,440.5	1,077.6	<0.001
TC (g/L)	14,626.0	3,679.7	981.5	<0.001

CPRS, continuous pit recirculation system; SEM, standard error of the mean; EC, electrical conductivity; BOD, biochemical oxygen demand; COD, chemical oxygen demand; SS, suspended solids; T-N, total nitrogen; T-P, total phosphate; TOC, total organic carbon; TC, total carbon.

**Table 3:** Chemical properties of the recirculation liquid based on the treatment process equipped within the continuous pit recirculation system

Chemical properties	Storage tank	Slurry pit	Outlet of slurry pit	Anoxic tank	SEM	p-value
EC (dS/m)	11.9	12.5	13.5	11.7	0.91	0.448
pH	7.8	7.9	7.8	7.9	0.05	0.057
BOD (mg/L)	1,379.9^ab^	1,620.3^a^	1,344.2^ab^	1,197.7^b^	97.69	0.032
COD (mg/L)	2,985.8	3,301.9	3,177.4	3,017.9	254.54	0.770
SS (mg/L)	2,407.9	3,377.8	2,652.9	2,478.3	295.09	0.095
T-N (mg/L)	1,008.5^b^	1,171.5^a^	1,134.4^ab^	1,054.2^ab^	47.05	0.008
T-P (mg/L)	106.1^b^	150.3^a^	109.6^b^	84.8^b^	10.58	0.001
NH_4_-N (mg/L)	429.2^c^	626.2^a^	577.4^ab^	471.4^bc^	33.55	0.000
TOC (g/L)	1,668.2	2,479.9	2,171.4	1,802.4	392.93	0.355
TC (g/L)	2,931.4	3,733.9	3,735.8	3,240.6	337.77	0.117

SEM, standard error of the means; EC, electrical conductivity; BOD, biochemical oxygen demand; COD, chemical oxygen demand; SS, suspended solids; T-N, total nitrogen; T-P, total phosphate; TOC, total organic carbon; TC, total carbon.

a–c Means in the same row with different superscripts differ significantly p<0.05.

**Table 4:** Comparison of odor intensity and atmosphere characteristics in the finishing pig house according to the application of continuous pit recirculation system

Items	Control	CPRS	SEM	p-value
Odor intensity^1)^				
Indoor	3.49	2.22	0.13	0.001
Exhaust	3.48	2.04	0.14	0.001
Outside	2.2	1.07	0.14	0.001
Indoor atmosphere				
Temperature (°C)	31.19	27.13	1.65	0.008
Humidity (%)	58.99	61.03	8.22	0.805
Wind velocity (m/s)	0.13	0.15	0.12	0.853
CO_2_ (ppm)	1,514.72	605.64	141.65	0.001
NH_3_ (ppm)	19.00	6.83	1.62	0.001
H_2_S (ppm)	0.54	0.11	0.14	0.008

CPRS, continuous pit recirculation system; SEM, standard error of the means.

1) Odor intensity was scored according to a 6-ladder whole number scale using the air dilution sensory method of the standard methods for the examination of odor [9] (0, undetectable; 1, barely detectable; 2, moderate; 3, strong; 4, very strong; 5, unbearable). Indoor, inner sampling point of finishing pig house (Figure 5); exhaust, exhaust fan sampling point of finishing pig house (Figure 5); outside, farm site boundary sampling point.

**Table 5:** Comparison of odorous material contents at various sampling points according to the application of continuous pit recirculation system

Odorous materials	Control^1)^			CPRS			SEM	p-value^2)^		
		
	Indoor	Exhaust	Outside	Indoor	Exhaust	Outside		S	L	S×L
NH_3_ (ppm)	19.98	17.73	4.23	6.38	6.73	0.51	1.18	0.000	0.000	0.001
H_2_S (ppm)	3.55	2.58	0.68	0.93	0.73	0.00	0.38	0.000	0.000	0.025
MM (ppb)	16.70	11.63	0.93	0.13	0.03	0.00	2.80	0.000	0.023	0.025
DMD (ppb)	0.88	0.90	0.00	0.00	0.00	0.00	0.07	0.000	0.000	0.000
TMA (ppb)	0.04	0.03	0.00	0.01	0.01	0.00	0.00	0.000	0.000	0.000
Phenol (ppb)	9.85	6.58	0.40	4.92	3.16	0.00	1.07	0.000	0.000	0.030
Indole (ppb)	5.38	5.35	0.35	2.45	1.78	0.00	0.42	0.000	0.000	0.000
Skatole (ppb)	7.38	3.83	0.45	2.51	0.78	0.00	0.54	0.000	0.000	0.001

CPRS, continuous pit recirculation system; SEM, standard error of the mean; MM, methyl mercaptan; DMD, dimethyl disulfide; TMA, trimethylamine.

1) Indoor, inner sampling point of finishing pig house (Figure 5, [d]); exhaust, exhaust fan sampling point of finishing pig house (Figure 5, [e]); outside, Farm site boundary sampling point.

2) S, the effect of recirculation system; L, the effect of analysis location; S×L, Interaction between recirculation and analysis location effect.
