# CORRIGENDUM

**DOI:** 10.5713/ab.21.0135C

**Published:** 2022-04-22

The followings are to be changed in Anim Biosci 2022;35(2):308–316.

Correct unit of TOC and TC in table 2 and 3 is mg/L (not g/L).

